# Measuring Problematic Internet Use, Internet Gaming Disorder, and Social Media Addiction in Young Adults: Cross-sectional Survey Study

**DOI:** 10.2196/27719

**Published:** 2022-01-27

**Authors:** Megan Moreno, Karyn Riddle, Marina C Jenkins, Ajay Paul Singh, Qianqian Zhao, Jens Eickhoff

**Affiliations:** 1 Department of Pediatrics University of Wisconsin-Madison Madison, WI United States; 2 School of Journalism and Mass Communication University of Wisconsin-Madison Madison, WI United States; 3 School of Medicine and Public Health University of Wisconsin-Madison Madison, WI United States; 4 Department of Biostatistics and Medical Informatics University of Wisconsin-Madison Madison, WI United States

**Keywords:** technology, young adults, addiction, social media, internet, video games, screening, surveillance, cross-sectional, survey, mobile phone

## Abstract

**Background:**

Digital technology use is nearly ubiquitous among young adults; this use provides both benefits and risks. The risks of technology use include maladaptive technology use or technology addiction. Several conceptualizations of these addictions have emerged, each with its own assessment tools. These conditions include problematic internet use (PIU), internet gaming disorder (IGD), and social media addiction (SMA). These conditions have been associated with health outcomes such as problematic alcohol use, sleep disorders, and mental illness. These maladaptive technology conditions have been most commonly studied in isolation from each other.

**Objective:**

The aim of this study is to examine PIU, IGD, and SMA together to better inform future research approaches and provider screening practices for young adults.

**Methods:**

This cross-sectional survey study was conducted using Qualtrics panel-based recruitment and survey hosting. We recruited US young adults aged 18-25 years. The survey assessed PIU, IGD, and SMA. Survey measures also included assessments of problematic alcohol use, sleep, depression, and anxiety. We evaluated the frequency of and overlap in positive screening scores among PIU, IGD, and SMA and modeled each condition using multivariate logistic regression. Finally, we calculated sensitivity and specificity, as well as the positive predictive value and negative predictive value of the screening tools using the most prevalent maladaptive technology type.

**Results:**

Our 6000 participants had an average age of 21.7 (SD 2.5) years. Of these 6000 participants, 3062 (51.03%) were female, 3431 (57.18%) were Caucasian, 1686 (28.1%) were in a 4-year college program, and 2319 (38.65%) worked full time. The mean PIU score was 3.5 (SD 3.1), and 53.58% (3215/6000) of participants met the criteria for PIU. The mean IGD score was 2.7 (SD 2.6), and 24.33% (1460/6000) of participants met the criteria for IGD. The mean SMA score was 7.5 (SD 5.7), and 3.42% (205/6000) met the criteria for SMA. Across all 3 maladaptive technology use diagnoses, there were varied associations with demographic variables and similar overlap with health outcomes. The sensitivity of PIU screening to detect IGD was 82% and to detect SMA was 93%, whereas the specificity and positive predictive value were much lower (37%-54% specificity; 6%-37% positive predictive value).

**Conclusions:**

This cross-sectional survey screened a large national sample of adolescents and young adults for PIU, IGD, and SMA to determine prevalence and overlap, demographic associations with each, and associations between these technology-related conditions and health outcomes. There was overlap across PIU, IGD, and SMA in some associated demographic variables and health outcomes. However, the patterns in the associated variables demonstrated unique qualities of each of these conditions.

## Introduction

### Background

Adolescents and young adults (AYAs) are often considered digital natives as they are growing up in a highly immersive technological society. Most US adolescents own their own personal smartphones, providing constant access to communication, information, and social networks [1]. AYAs’ frequent and consistent media use has both benefits and risks. Benefits include opportunities for creative expression and social support [2]. Risks include maladaptive technology use, including overuse and addiction. Following early efforts to conceptualize the meaning of maladaptive technology use, subsequent efforts have defined specific types of maladaptive technology use. In this paper, we focus on 3 common conceptualizations of maladaptive technology use: problematic internet use (PIU), internet gaming disorder (IGD), and social media addiction (SMA). These entities have most commonly been studied in isolation, limiting our ability to understand similarities and distinctions among these diagnoses. These 3 common conceptualizations of maladaptive technology use have commonalities in that they have often been associated with similar health conditions and commonly with mental health conditions [3-10]. Furthermore, although screening for problematic technology use is recommended by groups such as the American Academy of Pediatrics [11], screening efforts may be hampered if multiple assessments for different technologies are needed. Thus, an understanding of the intersection between types of maladaptive technology use and optimal screening tools is needed.

### Early Studies of Maladaptive Technology Use: Conceptualizing Mechanisms of Maladaptive Use

#### Overview

Efforts toward understanding maladaptive technology use began in the 1990s, with a focus on the internet and its overuse. Two initial conceptualizations were based on the existing Diagnostic and Statistical Manual of Mental Disorders, Fourth Edition disorders: substance abuse and dependency and pathological gambling [12,13]. From these early studies, 3 conceptualizations of maladaptive technology use emerged. One approach was broader, describing internet overuse as a general behavioral addiction [14,15]. A second approach was narrower, proposing a model that internet overuse should be more classified as an impulse control disorder with criteria defined as (1) maladaptive preoccupation with internet use characterized by either irresistible use or use that is excessive and longer than planned, (2) clinically significant distress or impairment, and (3) an absence of other explaining Axis 1 disorders [16]. A third approach proposed a cognitive behavioral model with focused attention on the impact of an individual’s thoughts on their development of problematic behaviors. This approach also separated internet overuse into *generalized* overuse or multidimensional overuse of the internet and *specific* overuse [17]. Specific overuse was defined as dependence on a specific function of the internet.

Since these initial conceptualizations, the field has changed both through additional evidence and conceptualizations of maladaptive technology use and on changing technology. Conceptualizing maladaptive use has moved forward as inclusive of constructs beyond overuse and now includes constructs to represent risky use and individual impairment. Furthermore, studies have introduced conceptualizations of specific addictions related to new technologies, such as video games and social media. In most cases, these conceptualizations all center on these behaviors as “pathological forms of normal and necessary behaviors” [18]. The 3 common areas of study in the current literature include PIU, IGD, and SMA.

#### Problematic Internet Use

A 2012 study developed a conceptual framework for PIU using empirical data and defined it as “internet use that is risky, excessive or impulsive in nature leading to adverse life consequences, specifically physical, emotional, social or functional impairment” [19]. The prevalence of PIU is estimated to be 4% among adolescents [20] and 4%-6% among young adults in college [21-23]. PIU has been associated with both social and health consequences, including poor academic performance, stress, and fewer positive health behaviors [22]. Longitudinal studies have also suggested bidirectional relationships between PIU and other mental health conditions, such as depression [3,24-26].

#### Internet Gaming Disorder

Maladaptive video game use is most commonly referred to as IGD, given that most video gaming occurs on the web. In 2013, the American Psychological Association proposed IGD as a disorder in need of further study by [27]. Defining characteristics of gaming addiction include spending increasing amounts of time preparing for, organizing, and actually gaming [28]. Another literature review found that internet gaming could be defined by a series of negative cognitions, including the following: (1) a consistent overvaluation of rewards, activities, and identities; (2) a need to adhere to self-applied rules for playing and finishing games; (3) an overreliance on playing video games as a means of enhancing one’s self-esteem; and (4) a means of social acceptance through either in-person gaming or web-based gaming. The estimated prevalence was described in a systematic review as 2% of children and adolescents affected by IGD, and the overall mean prevalence may reach 5.5% [29]. Negative consequences of IGD can include poor grades, academic problems, problematic alcohol use, depression, and negative self-esteem [8,10,29].

#### Social Media Addiction

SMA or addiction to social networking sites (SNSs) was defined in 2014 as “being overly concerned about SNSs, to be driven by a strong motivation to log on to or use SNSs, and to devote so much time and effort to SNSs that it impairs other social activities, studies or job, interpersonal relationships, and/or psychology health and well-being” [30]. Robust estimates of the prevalence of SMA are challenging, as most studies involve small and nonrepresentative samples of college students. In China, 2 studies reported 12% and 34% as the prevalence of SMA [7,31]. Studies on specifically Facebook addiction have reported prevalence rates between 1.6% and 8.6% [32,33]. SMA has been associated with sleep problems [34] and emotional problems, such as distress and depression symptoms [5,35].

### Previous Associations With Maladaptive Technology Use

A previous study examined the relationships between several behavioral addictions, including internet addiction, video game addiction, and Facebook addiction and the 5-factor model of personality [36]. The study found >20 correlations between the 7 behavioral addictions measured in the study, all of which were positive. However, across the 5 factors of personality, the 3 constructs in this study (PIU, IGD, and SMA) were grouped together only via a negative association with conscientiousness. Another study of adults examined IGD and SMA and several measures of mental health symptoms. They found correlations between maladaptive technology use and mental health symptoms and a weak interrelationship between IGD and SMA [4]. These studies suggest connections between these maladaptive technology use constructs, as well as ways in which each may be unique.

### Gaps in the Literature

Although tremendous strides have been made in the past several decades, several critical gaps remain in the literature. The divergent areas of focus for maladaptive technology use, including PIU, IGD, and SMA, mean that it remains unclear whether these diagnoses have similar associations with health outcomes. Although the literature has supported associations between PIU, IGD, and SMA and mental health conditions in particular, formal testing has not been conducted to evaluate the strength of these associations across these conditions. Furthermore, health care providers are increasingly called upon to screen for maladaptive technology use [11]. Thus, an understanding of how to approach initial screening and whether a single instrument or multiple scales are needed is important. This understanding is important to inform whether screening and treatment approaches need to be specific to certain technological platforms or broader.

### Study Purpose

Building on these gaps in the literature, this study has 2 goals. The first goal is to understand the overlap in prevalence, demographic factors, and health outcomes across PIU, IGD, and SMA. If these 3 diagnoses are truly distinct, we would expect little overlap in a study population when screening for all 3. Furthermore, we would expect to see unique patterns in the associated demographic factors with PIU, IGD, and SMA. Finally, we would expect to see distinct associations with health outcomes. For this study, we will focus on health outcomes that have been studied in previous work with maladaptive technology use, including problematic alcohol use, sleep, depression, and anxiety [24,37-40].

The second goal of this study is to consider our findings and their impact on screening options for health care providers. If these 3 diagnoses are unique, then individual screening for PIU, IGD, and SMA is warranted. If there is overlap, it may be possible to identify a screening instrument with optimal sensitivity to facilitate universal screening. Then, when a positive screen emerges for a given patient, individual screening tools can be provided to improve specificity.

The aim of this study is to examine PIU, IGD, and SMA to better inform future research approaches and provider screening practices for young adults.

## Methods

A web-based cross-sectional survey was conducted using Qualtrics panel-based recruitment and survey hosting. This study was approved by the University of Wisconsin institutional review board.

### Participants and Recruitment

Our goal was to achieve a purposeful national sample of young adults to complete a closed web-based survey. Compared with traditional survey approaches, such as in-person, phone, or mail recruitment, web-based survey panels offer broader reach and lower costs in data collection [41]. We selected the web-based survey platform Qualtrics for several reasons. First, although web-based survey platforms do not use weighting, previous studies have shown that web-based survey approaches using tools such as Qualtrics can achieve demographic attributes that are typically within a 10% range of their corresponding values in the US population [42]. Second, we sought to recruit a diverse sample of young adults both in and out of school settings, so recruiting using traditional approaches, such as at a college campus, would not achieve that goal. Third, there is a strong and growing literature on the use of Qualtrics to recruit a sample of young people in the United States, including studies on media [43,44].

The target population for this study was 18-25-year-olds who were US residents and English-speaking. We established parameters for Qualtrics to recruit a sample consistent with race or ethnicity representative of the US census population for 18-25-year-olds who could speak or read in English [42]. Using these eligibility parameters, a Qualtrics survey manager recruited young adult panel participants using email and texting. Potential participants were directed to the web-based survey website to obtain more information. Potential participants were provided information about the survey including information about the length and topic of the survey. Participants completed informed consent before beginning the survey.

### Survey Procedures

The survey was hosted on Qualtrics. Participants who provided consent were allowed to begin the survey. After informed consent was obtained, each measurement tool was provided on a single webpage. Demographic information was collected on a single webpage. Participants were allowed to skip questions or scales if they were uncomfortable or did not want to complete certain items. Participants could move backward and forward in the survey before submitting the results.

Participants were provided Qualtrics points as an incentive for survey completion. Survey results were delivered to the investigative team without identifying information.

### Survey Measures

Our 3 technology-related scales included assessments for PIU, IGD, and SMA.

#### Problematic Internet Use

The Problematic and Risky Internet Use Screening Scale (PRIUSS) [45] was developed based on the PIU conceptual framework [19]. The PRIUSS was validated for use among AYAs in English and Dutch [45-47]. The PRIUSS has 2 versions: a 3-item short screen designed as an initial screen to be followed by an 18-item full screen if the short screen is positive [48]. PIU was measured using the 3-item PRIUSS (PRIUSS-3) [46]. The scale asks participants to answer how often certain behaviors and experiences have happened in the past 6 months. For example, items include how often “do you feel irritated when you are away from the internet?” and “do you experience withdrawal when you are away from the internet?”

#### Internet Gaming Disorder

IGD was measured using the IGD Scale [49]. This 9-item scale asks participants to respond to whether they have had certain experiences in the past year. For example, items include “have there been periods when all you could think of was the moment that you could play a game?” and “have you felt unsatisfied because you wanted to play more?” Response options are yes and no. The cutoff for a positive score is ≥5 *yes* answers. The Cronbach α for this scale was .83.

#### Social Media Addiction

SMA was measured using the Bergan SMA Scale [37]. This scale has 6 items about the frequency of certain social media experiences over the past year. For example, statements include “You feel an urge to use social media more and more” and “You use social media in order to forget about personal problems.” Response options are on a 5-point Likert scale from *very rarely* to *very often*. The initial scale was conceptualized as a Facebook Addiction Scale, which was shown to have good psychometric properties [36] and was subsequently expanded to represent a broader SMA scale. The Cronbach α for this scale was .87.

#### Health Behavior and Conditions

Our health behavior and condition measures included problematic alcohol use, sleep, depression, and anxiety.

##### Problematic Alcohol Use

Problematic alcohol use has been associated with maladaptive use in previous work [9,10,40,50]. We measured problematic alcohol use using the Alcohol Use Disorders Identification Test-Concise [51,52]. This 3-item scale asks participants about their alcohol consumption habits. For example, items include frequency of drinking alcohol and number of alcoholic drinks consumed on a typical day. Responses are on a 5-point Likert scale, with higher scores indicating greater consumption. Scores were allotted from 0 to 4 on a Likert scale for each question. The cutoff used to indicate hazardous drinking was 4 points for men and 3 points for women.

##### Sleep

Robust literature, including a systematic review and a meta-analysis [53], links maladaptive technology use to sleep issues [50,54,55]. Sleep was measured using the Epworth Sleepiness Scale [56]. This 8-item scale asks participants how likely they are to doze off or fall asleep in certain situations. For example, items include sitting and reading or as a passenger in a car for an hour without a break. Response options are on a Likert scale from 0 to 3, from no chance of dozing off to a high chance of dozing off. Higher scores indicate increased sleepiness; a score ≥11 represents higher sleepiness. The scale defines mild sleepiness as scores 11-14, moderate sleepiness as scores 15-17, and severe sleepiness as scores 18-24.

##### Depression

Depression has consistently been associated with maladaptive technology use across multiple studies [3,5,7,8,24,52,57-59]. For this study, depression was measured using the Patient Health Questionnaire [60-62]. This 9-item scale asks participants how often they have experienced the given symptoms in the past 2 weeks. For example, items include “little interest or pleasure in doing things” and “feeling down, depressed or hopeless.” Response options used a 4-point Likert scale ranging from *not at all* to *nearly every day*. The scale defines mild depression as scores of 6-10, moderate depression as scores of 11-14, moderately severe depression as scores of 15-19, and severe depression as scores of 20 and above.

##### Generalized Anxiety Disorder

Anxiety has been linked to maladaptive technology use in previous work [3,8,57,58]. Anxiety was measured using the Generalized Anxiety Disorder–7 scale [63]. This 7-item scale asks participants how often they have experienced the given symptoms in the past 2 weeks. For example, items include “feeling nervous, anxious or on edge” and “trouble relaxing.” Response options used a 4-point Likert scale ranging from *not at all* to *nearly every day*. Scores for this scale include mild anxiety as scores 6-10, moderate anxiety as scores 11-15, and severe anxiety as scores of ≥16.

Demographic measures included age, gender, and race and ethnicity. We asked the participants about the highest grade they had completed, with answer options including some high school, high school graduate, some college, technical school or associate arts degree, college degree, some graduate school, completed a graduate degree, and others. We asked about current education or employment status as well (eg, part-time or full-time employment).

### Analysis

Statistical analyses were conducted using SAS software (SAS Institute Inc), version 9.4. All *P* values were 2-tailed, and *P*<.05 was used to indicate statistical significance. Descriptive statistics were summarized as frequencies and percentages or means and SDs.

Age was categorized as younger (18-20-year-olds) versus older (21-25-year-olds) AYAs. Race or ethnicity was categorized for analyses based on 2 goals. One goal was to leverage statistical power by collapsing some groups with smaller proportions of participants. This categorization led to several larger groups. Our second goal was to ensure that the groups identified in previous studies as at risk for problematic technology use were included in the analyses. Thus, we then reviewed previous studies of problematic technology use [7,64] to ensure that at-risk groups from these studies were included as distinct groups for analysis. Our final list of groups included Asian, Caucasian or White, Hispanic or Latino, African American, and others. The highest grade completed was dichotomized to include college education versus no college. Employment was dichotomized as any employment (full or part-time) versus not employed. Current schooling was dichotomized as in school (full or part-time) versus not in school.

To address our first study aim, we evaluated the frequency of and overlap in positive screening scores among PIU, IGD, and SMA. We calculated the proportions of participants who met the clinical criteria for PIU, IGD, and SMA using those scales’ validated score cutoffs.

To determine associations between demographic variables and maladaptive technology use diagnoses, as well as maladaptive technology use and health behavior and conditions, we used multivariate logistic regression.

Finally, we calculated the typical measures to assess the value of screening tests in clinical settings [65]. These typically include sensitivity, which is the ability of a test to correctly classify an individual as having a condition or the likelihood that a test is positive in a true positive case. These also include specificity, which is the ability of a test to correctly classify an individual as being without a condition or the likelihood that a negative test is a true negative. Positive predictive value (PPV) is the percentage of participants with a positive test to be positive cases, and negative predictive value (NPV) is the percentage of patients with a negative test who do not have the condition. An optimal screening test often relies on high levels of specificity and a high NPV to avoid missing a possible positive case. Screening tests are often followed by diagnostic tests, and an optimal diagnostic test often relies on high levels of sensitivity and PPV to correctly identify cases. We calculated the sensitivity and specificity of the screening tools using the most prevalent maladaptive technology type, defining that condition as the gold standard for this study.

## Results

### Overview

Our 6000 participants had an average age of 21.7 (SD 2.4) years. Of the 6000 participants, 3062 (51.03%) were female, 3431 (57.18%) were Caucasian, 1686 (28.1%) were in a 4-year college program, and 2319 (38.65%) worked full-time. Table 1 provides the demographic data.

**Table 1:** Demographic information of young adult participants recruited using Qualtrics panels (N=6000).

Variables		Values
Age (years), mean (SD)		21.7 (2.4)
**Gender, n (%)**		
	Female	3062 (51.03)
	Male	2841 (47.35)
	Other	91 (1.5)
**Race or ethnicity, n (%)**		
	Caucasian or White	3431 (57.18)
	Asian	304 (5.07)
	Hispanic or Latino	502 (8.37)
	Black or African American	793 (13.22)
	Other	957 (15.95)
**Highest grade completed, n (%)**		
	Some high school	101 (1.68)
	High school graduate	2149 (35.82)
	Some college	1682 (28.03)
	Technical school or associate arts degree	311 (5.18)
	College degree	1123 (18.72)
	Some graduate school	232 (3.87)
	Completed graduate degree	314 (5.23)
	Other	68 (1.13)
**Current education or employment, n (%)**		
	Part-time work	1853 (30.88)
	Full-time work	2319 (38.65)
	Part-time school	486 (8.1)
	Full-time school	1263 (21.05)

### Descriptive Data

Descriptive data for all measures are provided in Table 2. Descriptive data included mean scores across the maladaptive technology instruments. Findings included a mean score for problematic alcohol use of 2.6 (SD 2.6), mean score for depression of 9.4 (SD 7.2), mean score for anxiety of 8.4 (SD 6.1), and mean score for sleep of 9.07 (SD 5.2).

**Table 2:** Descriptive data from young adult participants.

Variables	Mean (SD; range)
PIU^a^	3.5 (3.1; 0-12)
IGD^b^	2.7 (2.6; 0-9)
SMA^c^	7.5 (5.7; 0-24)
Problematic alcohol use	2.6 (2.6; 0-12)
Depression	9.4 (7.2; 0-27)
Anxiety	8.4 (6.1; 0-21)
Sleep	9.1 (5.2; 0-24)

^a^PIU: problematic internet use.

^b^IGD: internet gaming disorder.

^c^SMA: social media addiction.

### Prevalence of PIU, IGD, and SMA

**Figure 1:**
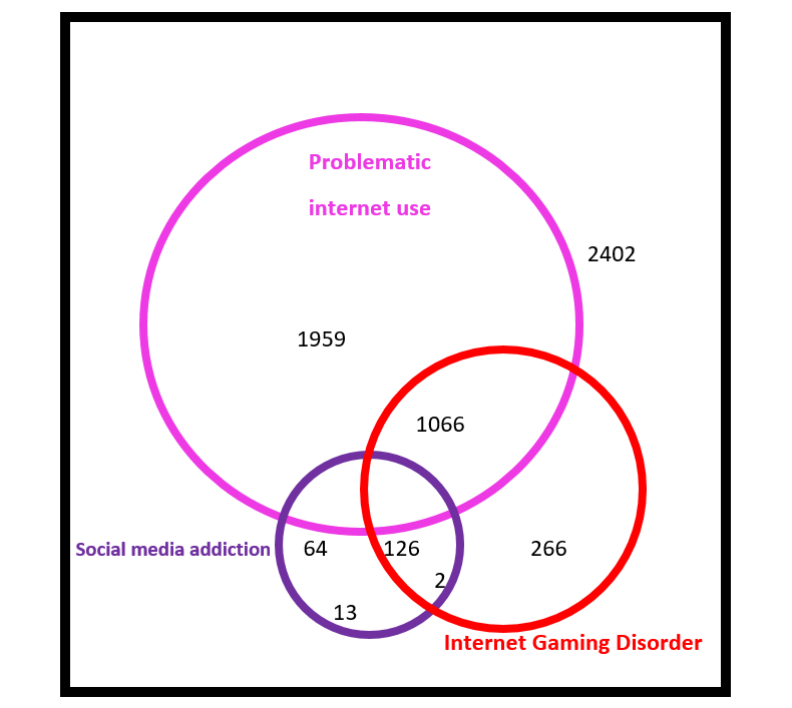
Overlap in screening rates for problematic internet use, internet gaming disorder and social media addiction among a young adult population.

For PIU, the mean PRIUSS score was 3.5 (SD 3.1), and 53.58% (3215/6000) of participants met the criteria for PIU. The mean IGD score was 2.7 (SD 2.6), and 24.33% (1460/6000) met the criteria for IGD. The mean SMA score was 7.5 (SD 5.7), and 3.42% (205/6000) met the criteria for SMA. Of the 6000 participants, 1959 (32.65%) met the criteria for only PIU, 266 (4.43%) met the criteria for only IGD, and 13 (0.22%) met the criteria for only SMA. Approximately 40.03% (2402/6000) did not meet the criteria for any of these diagnoses. Figure 1 represents the overlap in screening rates for PIU, IGD, and SMA.

### Associations With Demographic Variables Across PIU, IGD, and SMA

There were varied associations with demographic variables across all 3 maladaptive technology use diagnoses. There were no noted differences across younger and older AYAs in our study population for PIU, IGD, or SMA. Males were more likely to meet the criteria for PIU (odds ratio [OR] 1.2, 95% CI 1.0-1.4) and IGD (OR 2.9, 95% CI 2.5-3.5) than females. There was no gender association noted for SMA.

In evaluating race and ethnicity, Asian participants were more likely to meet the criteria for PIU than Caucasian participants (OR 1.7, 95% CI 1.3-2.4). Similar findings were present for IGD; Asian participants were more likely to meet the criteria for IGD than Caucasian participants (OR 1.6, 95% CI 1.2-2.2). Hispanic participants were also more likely to meet the criteria for IGD than non-Hispanic Caucasian participants (OR 1.3, 95% CI 1-1.7).

Higher educational levels were positively associated with PIU (OR 1.4, 95% CI 1.2-1.6) and SMA (OR 1.4, 95% CI 1-2) compared with lower educational levels. Being employed was positively associated with IGD (OR 1.3, 95% CI 1.2-1.5) than not being employed. Multimedia Appendix 1 illustrates these associations.

### Health Outcomes: Association With PIU, IGD, and SMA

For health behavior and condition variables, problematic alcohol use was positively associated with PIU, IGD, and SMA. Sleep issues were associated with PIU and IGD across all 3 sleep outcomes (mild, moderate, and severe sleepiness), whereas SMA was only associated with severe sleepiness (OR 4.6, 95% CI 2.9-7.2). Depression screening showed a similar pattern: there were associations with PIU and IGD across all categories of depression, although SMA was only associated with severe depression (OR 2.5, 95% CI 1.3-5.3). Anxiety also showed a similar pattern. Anxiety was associated with PIU and IGD across all categories of anxiety, and SMA was only associated with severe anxiety (OR 5.2, 95% CI 2.5-10.9). Multimedia Appendix 1 shows these associations.

### Screening for Maladaptive Technology Use

Given that PIU was the most prevalent maladaptive technology use diagnosis, we tested PIU as our defined gold standard in this study and its capacity to predict IGD and SMA. The sensitivity of PIU for detecting IGD was 82% (95% CI 80%-84%), and the specificity was 54% (95% CI 53%-56%). The PPV was 37% (95% CI 35%-39%), and the NPV was 90% (95% CI 89%-91%). Thus, the overall accuracy was 61% (95% CI 60%-62%).

The diagnostic accuracy of PIU for predicting SMA included sensitivity of 93% (95% CI 88%-96%), with a specificity of 47% (95% CI 46%-48%). The PPV was 6% (95% CI 5%-7%), and the NPV was 99% (95% CI 99%-100%). The overall accuracy was 48% (95% CI 47%-50%).

## Discussion

### Principal Findings

This cross-sectional survey screened a large national sample of AYAs for PIU, IGD, and SMA to determine prevalence and overlap, demographic associations with each, and associations between these technology-related conditions and health outcomes. There was overlap across PIU, IGD, and SMA in some associated demographic variables as well as health outcomes. However, the patterns in the associated variables demonstrated unique qualities of each of these conditions.

### Prevalence Differences in PIU Compared With IGD and SMA

Our first finding was that PIU was the most prevalent condition among our study population, and screening for PIU captured many of the participants who screened positive for IGD and SMA. Given that PIU is more nonspecific than IGD’s focus on video games and SMA’s focus on social media, this finding may seem logical. There is overlap in the emotions and behaviors asked in each of the scales for PIU, IGD, and SMA; however, the PRIUSS asked participants to consider their internet use more broadly. Placing these findings in the context of previous work, we can consider the early conceptualizations of problematic technology use. Our findings align with an early cognitive behavioral model with focused attention on the impact of an individual’s thoughts on their development of problematic behaviors. This approach separated internet overuse into *generalized* overuse or multidimensional overuse of the internet and *specific* overuse [17]. Specific overuse was defined as dependence on a specific function of the internet. We found that PIU, a more generalized condition, was the most prevalent of the 3 conditions and that IGD and SMA as more specific conditions were endorsed by smaller groups than PIU. These findings suggest that this generalized or specific model may provide insights into the mechanisms for the current state of problematic technology conditions.

### Prevalence of PIU Compared With Previous Studies

It is notable that prevalence rates for PIU in this study, using a brief screening tool, indicated that just over half of participants screened positive for PIU using the short PRIUSS-3 screen. Previous studies of college students using the longer PRIUSS-18 have suggested prevalence rates among 4-year college students of around 4% [21]. As these are 2 different but related instruments with different purposes, this is likely the main reason for the higher prevalence of screening at risk for PIU in this study. The PRIUSS-3 was designed to maximize sensitivity at the expense of specificity. As a screening tool for identifying individuals who would benefit from further screening, this tool has demonstrated 100% sensitivity and 69% specificity [48]. This design approach and screening purpose of the PRIUSS-3 is the most likely explanation for why the prevalence rates were higher compared with previous work.

A second consideration is whether these prevalence findings represent differences based on our broad study population of young adults. This study recruited a general population of young adults across the United States, a different approach than that of many previous studies that have focused on specific populations of college students [66-68]. This study’s focus on young adult populations may also explain the increased rates of IGD compared with previous studies.

A third possible consideration is whether our findings suggest an increasing prevalence of PIU within society over time. It can be argued that an increasing number of daily activities now occur on the web, such as shopping, viewing recipes, or learning new skills. Thus, the continued infringement of the internet into lives may lead to increasing reliance or dependence on web-based connections. However, given the stark differences in the prevalence for this study compared with others using the PRIUSS-18 and studying 4-year college student populations, this final consideration is not likely able to fully address or explain our findings.

### Correlations Between PIU, IGD, SMA and Health Outcomes

We found strong correlations between PIU and the mental illnesses of anxiety and depression in the 0.4 range, and similar findings for SMA were found. Correlations for gaming and anxiety and depression were lower, in the 0.27-0.34 range. For correlations with alcohol use, we found lower correlations with all our technology diagnoses; the highest correlation was with PIU at 0.21. For sleep, correlations were similar across PIU, IGD, and SMA, all in the 0.3 range. These findings support screening for depression and anxiety concomitantly with maladaptive technology use. These findings also illustrate subtle differences among PIU, IGD, and SMA, such as how participants who screened positive for IGD also demonstrated a lower correlation for anxiety compared with depression, which differed from the pattern for PIU and SMA. These findings should be explored in future studies.

### Limitations

This study’s results may not generalize beyond a study population of young adults recruited via Qualtrics. Recruiting from a web-based panel meant that we could designate the study population size and criteria; however, it limited our ability to assess external validity. However, the Qualtrics platform and panels have been used in previous technology-focused studies [44], and panel recruitment has been shown to closely approximate US populations [42]. A second limitation is that our measures were assessed using self-report and thus may be subject to social desirability bias and recall bias.

Given that our study is cross-sectional and not longitudinal, we cannot conclude the directionality of these correlations. It is possible that our study illustrates preferential web-based activities among young adults with depression or anxiety. Furthermore, our study did not test or posit mechanisms by which problematic technology use and health conditions may be related. Although previous literature has identified associations between problematic technology use and certain conditions, an exploration of the mechanisms to support these associations remains understudied. This area of inquiry will be important for future work.

Finally, our study represents a unique approach for measuring multiple maladaptive technology conditions in a single study; thus, placing the findings in the context of existing literature is more challenging because of this unusual approach. Future studies should consider longitudinal study designs incorporating more than one measure of maladaptive technology use to further understand the similarities and differences across these conditions.

### Implications

Despite these limitations, our study has several intriguing implications. The first implication is that the findings suggest that PIU, IGD, and SMA are more alike than different. We found some small differences with demographic factors, which may support group or cultural differences rather than different underlying mechanisms for these types of maladaptive technology use. Across our health outcomes, we found similar positive correlations across PIU and SMA with anxiety and depression, with IGD showing some small differences.

One clinical implication centers on our finding that PIU was the most common condition and that screening for PIU captured many participants who screened positive for IGD and SMA. Given that PIU was identified as having sensitivity to detect IGD and SMA at the levels of 82% and 98%, respectively, it is likely that screening for PIU may be a valid approach to detect concerns related to technology. Then, if the screen is positive, follow-up screening could include specific evaluations for IGD or SMA depending on the patient’s history. As the PRIUSS-3 is designed to optimize sensitivity, this tool is likely a reliable candidate for this type of initial screening. Furthermore, as the PRIUSS-3 includes 3 questions, this brief screening tool could be incorporated into clinical flows without undue patient or clinic staff burden.

Our study found that PIU, IGD, and SMA were also commonly associated with positive screens for depression and anxiety. Thus, another clinical implication is that for clinics that do not conduct routine depression and anxiety screening for all patients, a positive screen for PIU should also prompt a screen for depression and anxiety. Early and ongoing screening for PIU may be a potential approach for identifying young adults at risk for other mental illnesses, and it may prompt further evaluation.

### Conclusions

In conclusion, our study advances the understanding of PIU, IGD, and SMA by demonstrating their strong overlap in meeting diagnostic criteria and similarities in demographic risk factors. We also found similarities in health behavior and conditions across PIU and SMA in particular. If these 3 conditions were truly distinct, we would anticipate little overlap in a population when screening for all 3 maladaptive technology conditions. We can conclude that PIU, IGD, and SMA are more similar than different. Finally, we identified that an efficient screening approach might be to conduct initial screening for PIU, followed by technology-specific screening assessments and screening for depression and anxiety.

## Appendix

Multimedia Appendix 1 Associations between demographic factors and health outcomes with maladaptive technology conditions.

## Abbreviations

AYA: adolescent and young adult
IGD: internet gaming disorder
NPV: negative predictive value
OR: odds ratio
PIU: problematic internet use
PPV: positive predictive value
PRIUSS: Problematic and Risky Internet Use Screening Scale
SMA: social media addiction
SNS: social networking site
