# Supplementary material for: Measuring Problematic Internet Use, Internet Gaming Disorder, and Social Media Addiction in Young Adults: Cross-sectional Survey Study
Source: JMIR Public Health Surveill. 2022 Jan 27;8(1):e27719. doi: 10.2196/27719 (PMC8832277; doi:10.2196/27719)
Supplement: Multimedia Appendix 1 [file publichealth_v8i1e27719_app1.docx]

| Table S1. Associations between demographic factors and health outcomes with maladaptive technology conditions | | | | | | | | | | |
| --- | --- | --- | --- | --- | --- | --- | --- | --- | --- | --- |
| Variable | | Problematic Internet Use (PIU) | | | Internet Gaming Disorder (IGD) | | | Social Media Addiction (SMA) | | |
|  |  | Odds Ratio | 95% LCL* | 95% UCL | Odds Ratio | 95% LCL | 95% UCL | Odds Ratio | 95% LCL | 95% UCL |
| Age | 18-20 vs. 21+ | 1.10 | 0.97 | 1.24 | 0.92 | 0.80 | 1.06 | 0.91 | 0.64 | 1.26 |
| Gender | Male vs. Female | **1.17** | **1.02** | **1.35** | **2.99** | **2.55** | **3.51** | 1.42 | 0.98 | 2.04 |
| Race | Asian vs. White | **1.75** | **1.30** | **2.37** | **1.59** | **1.16** | **2.17** | 1.72 | 0.84 | 3.28 |
|  | Hispanic vs. White | 0.92 | 0.73 | 1.15 | **1.31** | **1.00** | **1.69** | 0.88 | 0.44 | 1.65 |
|  | Black vs. White | 0.93 | 0.77 | 1.14 | 1.20 | 0.96 | 1.50 | 1.52 | 0.94 | 2.44 |
|  | Other vs. White | 0.91 | 0.76 | 1.10 | 1.21 | 0.99 | 1.49 | 1.04 | 0.64 | 1.68 |
| Education | College or Higher vs. Not | **1.40** | **1.22** | **1.61** | 1.12 | 0.96 | 1.31 | **1.42** | **1.00** | **2.00** |
| Employed | Yes vs. No | 0.93 | 0.80 | 1.07 | **1.30** | **1.09** | **1.55** | 1.03 | 0.68 | 1.59 |
| School | Yes vs. No | **1.20** | **1.03** | **1.38** | 0.94 | 0.79 | 1.11 | 0.80 | 0.52 | 1.22 |
| Sleep | Average vs. Normal | **1.58** | **1.33** | **1.88** | **1.63** | **1.31** | **2.01** | 0.57 | 0.27 | 1.13 |
|  | Excessive situational vs. Normal | **2.49** | **2.16** | **2.87** | **2.00** | **1.69** | **2.38** | 0.98 | 0.61 | 1.57 |
|  | Excessively vs.  Normal | **3.54** | **2.82** | **4.47** | **4.22** | **3.38** | **5.28** | **4.59** | **2.99** | **7.17** |
| Alcohol | Drinking problem vs.  none | **1.21** | **1.06** | **1.37** | **1.26** | **1.09** | **1.44** | **1.83** | **1.31** | **2.58** |
|  | Controlled drinking  vs. none | 1.03 | 0.58 | 1.82 | 0.73 | 0.30 | 1.54 | 0.75 | 0.04 | 3.90 |
| Depression | Mild risk vs. none | **1.98** | **1.67** | **2.35** | **1.88** | **1.48** | **2.38** | 0.79 | 0.36 | 1.73 |
|  | Moderate risk vs. none | **3.42** | **2.81** | **4.16** | **2.98** | **2.34** | **3.82** | 1.03 | 0.48 | 2.26 |
|  | Severe risk vs. none | **4.10** | **3.30** | **5.09** | **3.72** | **2.88** | **4.81** | **2.54** | **1.27** | **5.30** |
| Anxiety | Mild risk vs. none | **1.85** | **1.56** | **2.18** | **1.91** | **1.53** | **2.40** | 1.05 | 0.49 | 2.25 |
|  | Moderate risk vs. none | **2.13** | **1.75** | **2.60** | **2.22** | **1.74** | **2.83** | 1.24 | 0.58 | 2.72 |
|  | Severe risk vs. none | **2.15** | **1.70** | **2.72** | **2.00** | **1.53** | **2.63** | **5.20** | **2.56** | **10.99** |
| *LCL: Lower confidence level, UCL: Upper confidence level, bold text indicates *P*<0.05 | | | | | | | | | | |
